# Biochar and Activated Carbon as Formaldehyde Scavengers in Particleboards: A Technical and Environmental Assessment

**DOI:** 10.3390/ma19214571

**Published:** 2026-10-29

**Authors:** Hossein Mazaheri, Papiya Bhowmik, Brijesh Mainali, Reza Hosseinpourpia

**Affiliations:** 1 Department of Micro and Nanotechnology, Middle East Technical University, Ankara 06800, Türkiye; hossein.shadbad@metu.edu.tr; 2 Department of Forestry and Wood Technology, Linnaeus University, 351 95 Växjö, Sweden; papiya.bhowmik@lnu.se; 3 Department of Built Environment and Energy Technology, Linnaeus University, 351 95 Växjö, Sweden; brijesh.mainali@lnu.se; 4 Linnaeus University Centre for Competitive Timber Structures, Linnaeus University, 351 95 Växjö, Sweden; 5 College of Forest Resources and Environmental Science, Michigan Technological University, Houghton, MI 49931, USA

**Keywords:** mechanical properties, thickness swelling, urea–melamine–formaldehyde, greener particleboard, life cycle assessment

## Abstract

**Highlights:**

- AC and low-dose BC reduced formaldehyde emissions; BC2 slightly increased emissions.
- BC1 increased internal bond strength by 18%, while BC0.5 increased MOR by 33%.
- AC2 reduced 24 h thickness swelling by approximately 21%.
- BC incorporated particleboards had lower impacts than AC in most assessed environmental categories.
- BC2 gave approximately 14% lower annualized impacts under the assumed service-life model.

**Abstract:**

Activated carbon (AC) and biochar (BC) were incorporated into urea–melamine–formaldehyde (UmF) adhesive at 0.5, 1.0, and 2.0 wt% of adhesive solids to investigate their effects on particleboard performance. This study directly compares AC and BC in the same UmF-bonded particleboard system, combining measurements of formaldehyde release and technical performance with life cycle assessment and exploratory performance-based annualization. Compared with the control, boards containing 1.0% BC exhibited approximately 18% higher mean internal bond strength and 11% lower mean formaldehyde release, while 0.5% BC exhibited approximately 33% higher mean modulus of rupture. AC at 2.0% reduced mean 24 h thickness swelling by approximately 21%. Increasing BC loading to 2.0% did not improve formaldehyde control and slightly increased mean release. These experimental trends indicate that additive selection involves trade-offs rather than uniform improvements across properties. Baseline LCA results showed small differences in impacts per cubic metre of board. Under the assumed service-life model, equal weighting of the selected technical properties, and an equal-raw-impact assumption, BC2 yielded approximately 14% lower annualized impacts than the control. This modeled reduction does not establish an actual extension of service life. The combined assessment highlights the need to distinguish measured performance from assumption-dependent environmental rankings when selecting carbon-based additives.

## 1. Introduction

The wood-based panel industry relies extensively on formaldehyde-based adhesives, particularly urea–formaldehyde (UF) and urea–melamine–formaldehyde (UmF) adhesives, due to their high reactivity, cost-effectiveness, and ability to form durable cross-linked networks through condensation polymerization [1,2,3,4]. However, their application is associated with the emission of free formaldehyde during both production and service life, raising serious environmental and health concerns. Formaldehyde is a well-known indoor air pollutant and has been classified as a human carcinogen by the International Agency for Research on Cancer (Lyon, France) [5]. Exposure to indoor air pollutants, including volatile organic compounds such as formaldehyde, has been linked to significant adverse health effects and contributes to millions of cases of illness and mortality worldwide [6].

To address these concerns, extensive research has focused on developing formaldehyde-free or bio-based adhesives derived from renewable resources such as lignin, tannins, starch, and chitosan, with recent advances demonstrating improved adhesive performance through chemical modification strategies [7,8,9,10,11,12,13,14]. Despite these promising developments, bio-based adhesives still face limitations in industrial scalability, hindering their widespread adoption in large-scale panel production [15].

An alternative, more readily applicable strategy is to incorporate formaldehyde scavengers into conventional adhesive systems. In this approach, porous materials are introduced into the adhesive matrix to adsorb free formaldehyde or restrict its diffusion pathways, thereby reducing emissions without fundamentally altering the adhesive chemistry [16]. The performance of the scavengers is strongly governed by their pore size, structure and surface chemistry. Formaldehyde molecules, with an approximate kinetic diameter of 4 Å, are preferentially adsorbed within microporous structures. However, pore-size distribution can govern adsorption–desorption dynamics and long-term retention efficiency [3,17,18]. While micropores enhance adsorption capacity, larger pores may facilitate molecular diffusion and desorption rates, potentially reducing long-term capture efficiency. Although previous studies have investigated formaldehyde adsorption by porous materials under simplified conditions, such as direct adsorption of formaldehyde onto isolated adsorbents, their behavior when incorporated into the more complex and reactive adhesive–wood matrix of particleboards remains insufficiently explored.

Mineral-based materials, including zeolites and silica, have been investigated for formaldehyde control. Becker et al. [19] examined gaseous formaldehyde adsorption on Y zeolites, whereas Candan and Akbulut [20] reported reduced formaldehyde content in UF-bonded particleboards incorporating nanosilica. Activated carbon (AC) has also been previously reported as an effective scavenger due to its high specific surface area and well-developed microporous structure, which enable efficient adsorption of small molecules such as formaldehyde. It can significantly reduce formaldehyde emissions in urea–formaldehyde adhesives while maintaining or even enhancing certain mechanical properties of wood-based panels [21,22]. In addition, advanced carbon-based materials, including functionalized graphene and nano-sized carbons, have demonstrated high adsorption capacities owing to their large surface areas and reactive functional groups, such as hydroxyl, carboxyl, and carbonyl groups, which can interact with formaldehyde molecules [23,24]. Biochar (BC) has also been increasingly investigated as a functional filler in polymer and composite systems, where its porous structure and surface chemistry can enhance mechanical performance and interfacial interactions [25,26]. The porous network of biochar can promote mechanical interlocking by allowing polymer or adhesive penetration into its internal structure, while surface functional groups, when present, may further contribute to filler–matrix interactions [25,26]. These mechanisms improve stress transfer across the interface and enhance final composite performance [25]. These characteristics suggest that, in addition to formaldehyde adsorption, biochar may influence the curing behavior of the adhesive due to its carbonaceous nature, which can promote more uniform heat distribution during hot pressing and potentially result in a more homogeneous curing process. Furthermore, the use of biochar may support circular economy principles by valorizing waste biomass, providing additional motivation to investigate its potential as a formaldehyde scavenger in particleboard production [15].

Although AC and BC are both carbonaceous porous materials, their structural characteristics and production processes differ considerably. AC generally exhibits a highly developed microporous structure and high specific surface area, favoring adsorption, whereas BC can contain a broader pore-size distribution and surface functionalities that may influence both adsorption and interactions with the adhesive matrix. These differences may result in distinct effects on formaldehyde emission, mechanical performance, and environmental impacts.

Previous studies demonstrate that the effects of carbon-based additives depend on loading and composite system. Kawalerczyk et al. [22] incorporated coconut-shell activated carbon into UF-bonded particleboards at loadings of 0.25–1.5%. Additions of 0.75–1.0% improved internal bond strength and bending properties, whereas 1.0–1.5% reduced formaldehyde content by approximately 16%. Increasing the loading to 1.5% did not further improve mechanical performance. In a different system, Das et al. [25] investigated biochar contents of 6–30 wt% in wood/polypropylene composites and reported improved tensile and flexural properties as biochar content increased to 24 wt%. These findings support the potential of carbonaceous fillers, but the thermoplastic matrix and substantially higher biochar contents limit direct comparison with low-dose adhesive modification. The present study extends this research through a direct comparison of AC and BC at matched loadings in UmF-bonded particleboards, jointly assessing formaldehyde release, mechanical properties, thickness swelling, and environmental impacts.

Despite the increasing interest in carbon-based scavengers, a practical comparison between activated carbon and biochar in particleboard applications, particularly considering both mechanical performance and environmental impacts, remains limited. Several studies have evaluated the environmental impacts of wood-based panels using Life Cycle Assessments (LCAs) [27,28,29]. However, these assessments are typically based on mass or volume functional units and do not account for variations in service life due to mechanical performance, which may lead to incomplete or misleading sustainability evaluations.

Therefore, the aim of this study is to comparatively evaluate activated carbon and biochar as formaldehyde scavengers in UmF-bonded particleboards. The effects of these additives on mechanical properties, thickness swelling, and formaldehyde emissions are systematically investigated. In addition, LCA is conducted to assess the environmental implications of incorporating these materials. An exploratory performance-based normalization model is also applied to examine how assumed relationships between technical properties and service life influence annualized environmental impacts. This model supports comparison among formulations rather than predicting actual durability.

## 2. Materials and Methods

### 2.1. Materials

Fresh wood chips with particle sizes of 2 to 7.2 mm, as well as urea–melamine–formaldehyde (UmF) adhesive (68% solids) and its hardener (ammonium sulphate with 50% solids) were provided by IKEA Industry, Sweden (Hultsfred, Sweden). Biochar (BC) was produced from hardwood chips at approximately 800 °C for 30–45 min without a separate activation step. It was subsequently ground and sieved through a 150-mesh sieve to obtain particles smaller than 100 µm. Activated carbon (DARCO^®^, 20–40 mesh particle size, granular) was purchased from Sigma-Aldrich (St. Louis, MO, USA). The Brunauer–Emmett–Teller (BET) surface areas of AC and BC were 639 m^2^/g and 423 m^2^/g, respectively.

### 2.2. Particleboard Manufacturing

Single-layer particleboards with a thickness of 12 mm and a target density of 620 kg/m^3^ were produced with oven-dried chips with an average moisture content of 2%. The UmF adhesive was applied at 10% based on the oven-dry mass of the wood particles, while the hardener was added at 4% of the dry adhesive solid mass. The BC and AC were initially dispersed into the adhesive system at three levels (0.5%, 1.0%, and 2.0% by weight of adhesive solids), and the resulting mixture was sprayed onto the wood particles using a laboratory spray drum to ensure homogeneous distribution. The resinated particles were then formed into mats and hot-pressed at 180 °C using a pressing time of 10 s per millimetre of panel thickness. After pressing, the boards were conditioned in a climate chamber for seven days before testing. The control panels were prepared using only UmF adhesive. The labeling of the manufactured boards is shown in Table 1.

### 2.3. Characterisation

The AC and BC were investigated using a Fourier Transform Infrared (FTIR) spectrometer in attenuated total reflectance mode (PerkinElmer, High Wycombe, UK). The FTIR spectra were recorded over the wavenumber range of 4000 to 750 cm^−1^ with a spectral resolution of 4 cm^−1^ and 32 scans. The curing performance of the adhesives was characterized using a Differential Scanning Calorimetry (DSC) TA instrument (DSC25). The curing kinetics were analyzed by exposing ~6 mg of adhesives in a sealed aluminum crucible with a perforated crucible lid over a temperature range of 25 to 275 °C at different heating rates of 5, 10, 15, and 20 °C min^−1^ under a pure nitrogen atmosphere.

### 2.4. Technical Properties of Particleboards

Internal bonding strength (IB) was measured on ten (10) replicates in accordance with EN 319 [30] on samples with dimensions of 50 × 50 × 12 mm^3^ (length × width × thickness). The bending properties of the panels were evaluated by means of modulus of rupture (MOR) and modulus of elasticity (MOE) by three-point flexural testing of five (5) samples measuring 200 × 50 × 12 mm^3^ (length × width × thickness) in accordance with EN 310 [31]. The mechanical properties were tested using an MTS 10 kN universal testing machine. Thickness swelling (TS) was determined following EN 317 [32] using ten replicates per panel type with dimensions of 50 × 50 × 12 mm^3^ (length × width × thickness) after 2 and 24 h of water immersion. Statistical analysis of the mechanical properties and thickness swelling results was performed using single-factor ANOVA in Microsoft Excel, comparing scavenger-containing panels versus the control ones.

The formaldehyde emissions from the particleboards were determined using a GA-6000 gas analyzer (GreCon GmbH, Alfeld, Germany) following SS-EN ISO 12460-3:2023 [33]. Two samples were measured, and each particleboard sample was placed in a controlled setup where the air above the board was continuously bubbled through a series of four washing bottles containing distilled water. Each bottle collected formaldehyde for one hour, resulting in a total sampling time of four hours. After the experiment, the formaldehyde concentration in each bottle was measured, and the total emission from the sample was calculated by summing the values from all four bottles.

Normality was assessed using Shapiro–Wilk tests on residuals from separate one-way models with formulation as the factor. Homogeneity of variance was assessed using median-based Levene tests. Both checks used the same specimen subsets as the reported means, with a significance threshold of α = 0.05.

### 2.5. Environmental Assessment of Particleboards

The environmental performance of the manufactured particleboard panels was evaluated through Life Cycle Assessment (LCA). The methodology followed the ISO 14040/44 standards [34,35]. The LCA calculations were performed using Microsoft Excel for Microsoft 365 (Microsoft Corporation, Redmond, WA, USA).

#### 2.5.1. Goal, Scope, and Functional Unit

**Figure 1:**
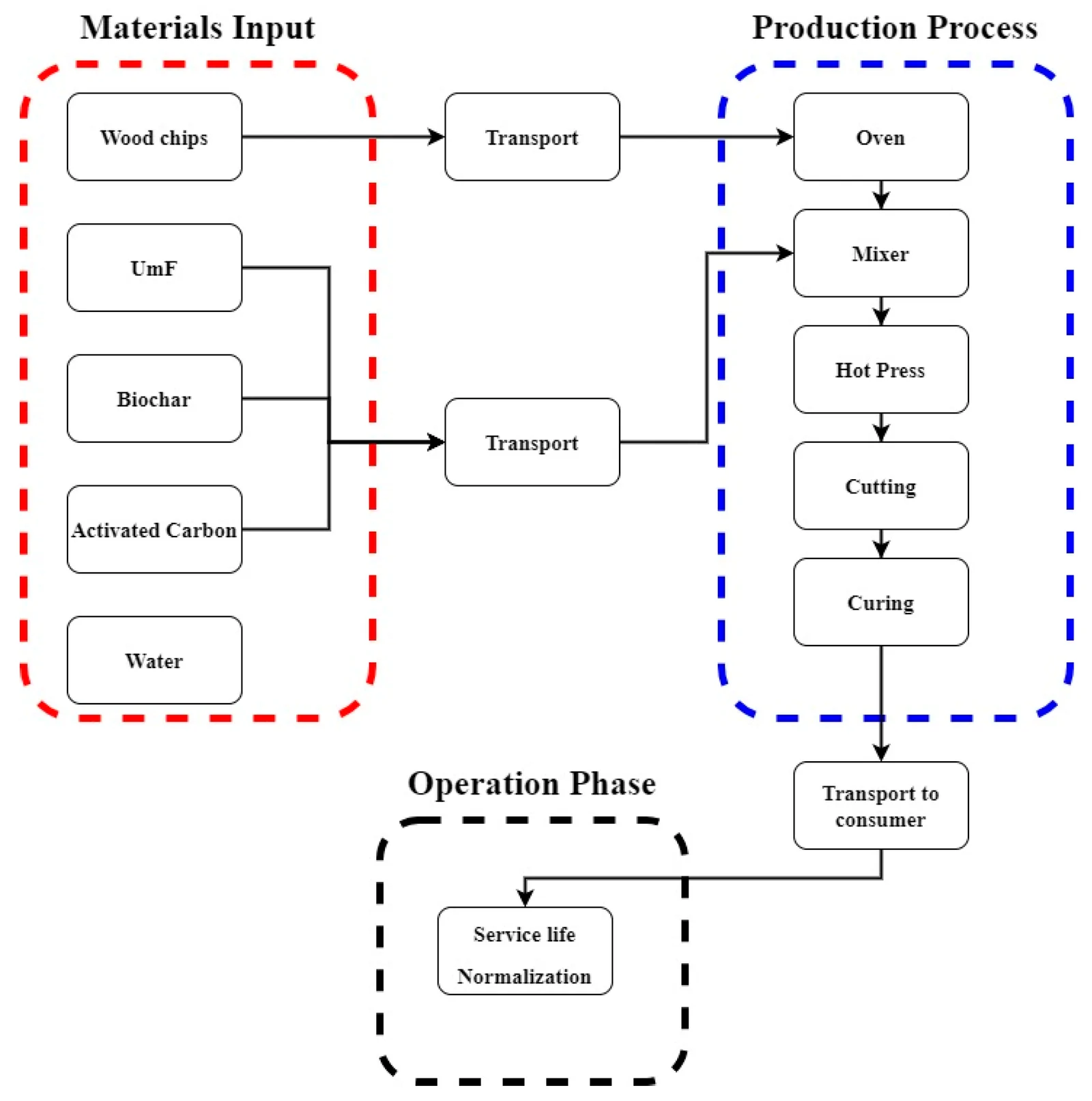
System boundaries of the life cycle assessment for particleboards using activated carbon and biochar scavengers.

The goal of this study was to assess the environmental impacts of particleboards containing AC and BC scavengers compared to conventional panels (Figure 1). A cradle-to-use analysis was carried out, and the functional unit was defined as 1 m^3^ of particleboard. No additional credit was assigned for biogenic carbon stored in the biochar incorporated into the particleboards. Although biochar can retain biomass-derived carbon during the use phase, the duration of storage and its eventual release depend on subsequent end-of-life management, which falls outside the cradle-to-use system boundary adopted in this study. Long-term carbon sequestration was therefore not quantified, and no claim of permanent carbon removal is made.

The assessment considered additive production impacts and explored how differences in board properties could influence annualized impacts under an assumed service-life relationship. Previous studies have examined particleboard deterioration and mechanical performance under outdoor exposure [36,37]. However, these studies do not validate a proportional relationship between the selected properties and the service life of the present indoor-use particleboards. Equations (1)–(5) were formulated for this study to construct a dimensionless comparative performance index using IB, MOR, and TS. The formulation follows the general principle of weighted additive aggregation of normalized indicators [38]. IB and MOR were expressed relative to the control, whereas the TS ratio was inverted so that higher values consistently indicate better performance.

For this exploratory comparison, a dimensionless performance index (PI) was defined by combining normalized internal bond strength, modulus of rupture, and inverse thickness swelling, as follows:

PI = W_IB_S_IB_ + W_MOR_S_MOR_ + W_TS_S_TS_ (1)

S_IB_ = IB_Sample_/IB_Control_ (2)

S_MOR_ = MOR_Sample_/MOR_Control_ (3)

S_TS_ = TS_Control_/TS_Sample_ (4)

W_IB_ + W_MOR_ + W_TS_ = 1 (5)

where S denotes a dimensionless property ratio and W denotes its weighting factor. IB, MOR, and TS represent internal bond strength, modulus of rupture, and thickness swelling, respectively. Equal weights were assigned to the three properties (W_IB_ = W_MOR_ = W_TS_ = 1/3). The TS ratio was inverted because lower swelling indicates better dimensional stability. A reference service life (RSL) of 30 years was assumed for the control board, and the assumed service life of each modified board was calculated as:

Assumed service life (SL) = PI × RSL(6)

This simplification is applied only to enable comparison of the panels among themselves, and for exact values, more detailed studies are required.

#### 2.5.2. Life Cycle Inventory

The life cycle inventory (LCI) was compiled using experimental data and secondary data from the Ecoinvent database (version 3.9). Primary data for laboratory-scale particleboard production, including material and energy consumption, were obtained from the experimental manufacturing process. Background data for material production, electricity generation, water supply, and transportation were obtained from Ecoinvent using the allocation, cut-off by classification system model. The Ecoinvent datasets used for the main inputs and processes are summarized in Table 2. A suitable biochar production dataset was not identified in the Ecoinvent version used in this study. Therefore, the charcoal dataset was used as a proxy. The biochar used experimentally was produced from hardwood chips at approximately 800 °C for 30–45 min, without a separate activation step. The proxy does not necessarily capture the specific feedstock, operating conditions, energy recovery, or allocation of environmental burdens between biochar and energy at the actual production facility. Its use therefore introduces uncertainty into the estimated impacts of the BC-containing boards. A sensitivity analysis was conducted by independently increasing and decreasing the production impacts assigned to BC and AC by 50%, while keeping additive dosages and all other inventory inputs unchanged. These inputs were selected because additive type and dosage constitute the main inventory differences among the board formulations. The ±50% range was used as an exploratory scenario range rather than a measured uncertainty interval. Changes in each impact category were calculated relative to the baseline result for the corresponding formulation.

Transport distances were estimated based on the geographical origin of the supplied materials and their delivery to Linnaeus University, Växjö, Sweden. Wood chips, UmF adhesive, and hardener were supplied by IKEA Industry in Hultsfred, Sweden, located approximately 110 km from Växjö. Activated carbon (AC) was procured from Sigma-Aldrich (Merck KGaA, Darmstadt, Germany). As the exact production site was not specified, Darmstadt, Germany, was considered as the origin, corresponding to an estimated road transport distance of approximately 850 km to Växjö. Biochar (BC) was locally obtained in Växjö, Sweden, and was therefore assigned a negligible transport distance.

The life cycle impact assessment (LCIA) was performed using the CML-IA baseline method, considering impact categories including global warming potential (GWP), human toxicity (cancer and non-cancer effects), abiotic resource depletion, acidification potential, eutrophication potential, photochemical oxidation, and freshwater aquatic ecotoxicity. These indicators were selected to evaluate the potential environmental impacts associated with the production and modification of UmF adhesive and particleboard manufacturing.

## 3. Results and Discussion

### 3.1. FTIR Spectra of Activated Charcoal and Biochar

**Figure 2:**
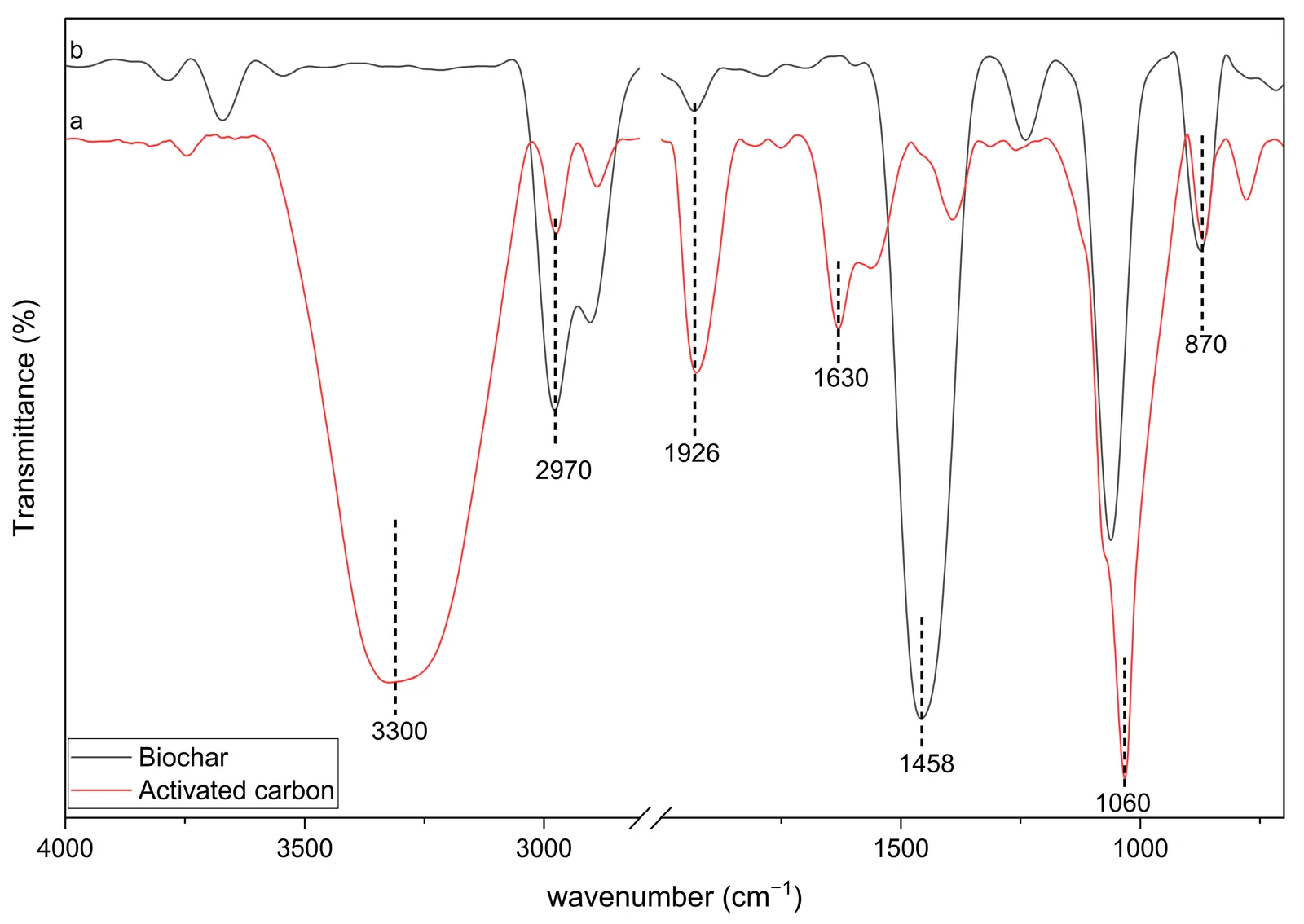
FTIR spectra of (a) activated carbon and (b) biochar.

The FTIR spectra of activated carbon and biochar showed distinct differences in the chemical compositions and relative intensities of the functional groups (Figure 2). Activated charcoal showed a broad, intense band at approximately 3300 cm^−1^, whereas this feature was weak in the biochar spectrum. This band is assigned mainly to the stretching vibration of O-H groups associated with phenolic and alcoholic hydroxyls and physically adsorbed water [39,40]. The absorption at approximately 2970 cm^−1^ is attributable to asymmetric stretching of aliphatic C-H bonds, particularly terminal CH_3_ groups, with a possible contribution from CH_2_ groups [39,41]. Activated charcoal showed a distinct band at approximately 1926 cm^−1^, while biochar showed only a weak peak, which can be conservatively described as an absorption associated with highly conjugated carboxylic acids, ketones, or esters, or as an overtone band. Activated charcoal also showed a pronounced band at approximately 1630 cm^−1^. In carbonized lignocellulosic materials, this region is commonly associated with aromatic C=C skeletal stretching, although conjugated C=O groups, quinone-like structures, and H–O–H bending from adsorbed water can overlap at nearly the same position [40,41]. Studies of ash-rich and wood-derived biochars have linked the paired bands at approximately 1416–1450 and 870–875 cm^−1^ to carbonate minerals, particularly CaCO_3_ [42].

### 3.2. Curing Performance of Adhesive

**Figure 3:**
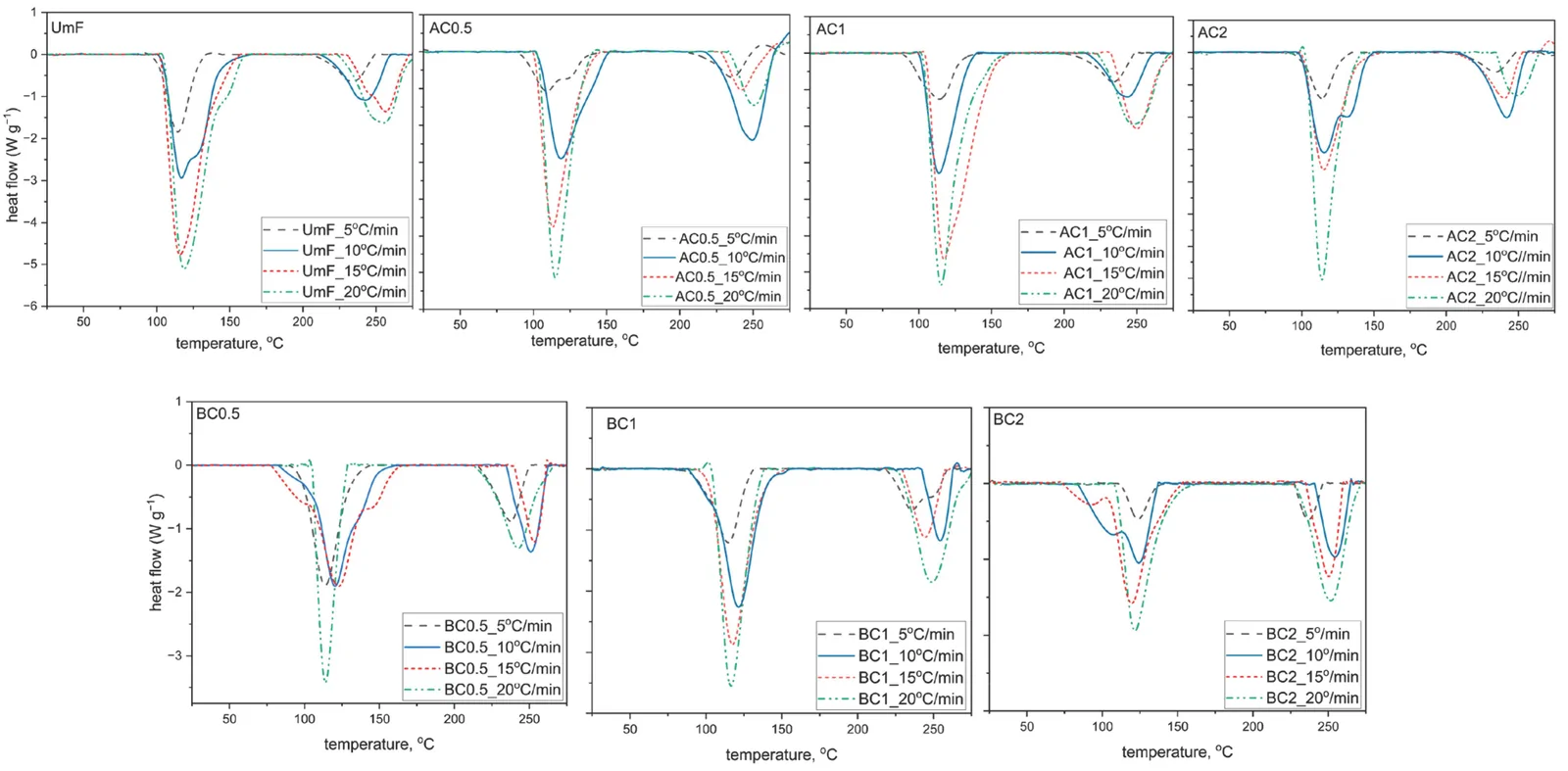
DSC thermograms of activated carbon- and biochar-reinforced UmF adhesive.

**Figure 4:**
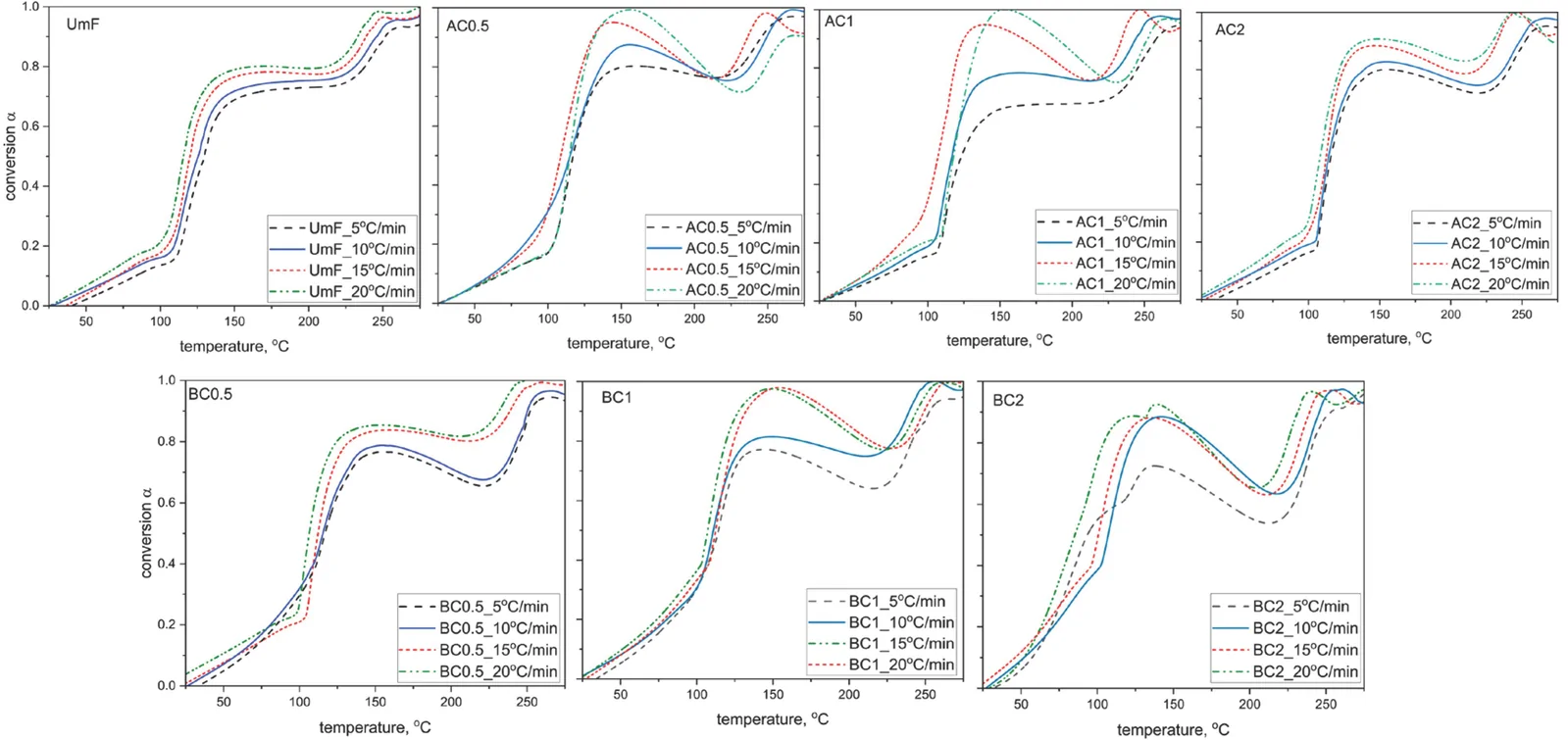
The conversion alpha of the adhesives at temperatures ranging from 25 °C to 275 °C.

The heat-flow curves showed a strong low-temperature exothermic event, with its minimum generally near 110–125 °C, followed by a second event near 235–255 °C. As shown in Figure 3, addition of AC and BC modified the principal thermal response of the UmF in a formulation-specific manner. The formulas containing AC and BC1 illustrated that the first exothermic peaks constantly increased with increasing heating rate. This is similar to the peaks observed in pure UmF at the first event, and reflects the scan-rate effect on the curing performance of the adhesive formulas, where increasing the programmed heating rate from 5 to 20 °C min^−1^ generally shifts the low-temperature exotherm to higher temperatures and increases its peak intensity. This is expected in non-isothermal DSC as the sample has less time to react at each temperature as the ramp accelerates [43]. The intensity of the peaks in the second event (235–255 °C) did not follow specific patterns as a function of heating rate. The first event is mainly consistent with the principal acid-catalyzed condensation/crosslinking of the UmF adhesive, while the second event in apparent conversion could reflect residual condensation, rearrangement, or degradation of the cured resin, as well as thermal contributions from the wood-derived carbon [43,44]. This pattern was also reflected in the curing kinetics of adhesive formulas, as indicated by conversion α (Figure 4). An α value of 1 is assumed to correspond to a fully cured adhesive [45]. The pure and modified UmF adhesive formulas presented two-step curing patterns. The slope of the isoconversional lines during the first step was influenced by additive load and type, where formulas containing AC showed steadier slopes from 100 °C to 150 °C than those containing BC. Increasing the heating rate also increased the slope of the isoconversional lines, which might be attributed to the formation of chemical bonds in the adhesive formulas [45]. Except for the pure UmF, which showed a gradual increase in conversion α at elevated temperatures, corresponding to the progression of curing, most AC- and BC-containing formulas demonstrated slight declines between around 150 °C and 220 °C and then increased until α = 1. This behavior might be due to the realignment of the polymer and reorganization of the methylene bridge with formaldehyde in the presence of AC and BC [21].

### 3.3. Mechanical Properties

The internal bonding (IB) strength results of the particleboard panels, along with their statistical significance, are presented in Table 3. Among all panels, BC1 resulted in a great improvement in IB, with about 18% higher value than the control. This enhancement was statistically significant (*p* ≤ 0.05), indicating a reliable improvement in inter-particle bonding. The BC2 and AC1 panels also showed moderate increases in IB (10.42% and 9.26%, respectively), while these values were statistically insignificant compared to the control samples (*p* > 0.05). The addition of AC at 0.5% and 2.0% reduced IB strength. The significantly higher IB of BC1 may be associated with the particle characteristics and distribution of biochar within the adhesive matrix. A possible explanation is that the BC particles promoted interfacial contact between the wood particles and adhesive; however, particle dispersion was not directly characterized in this study. Furthermore, the porous structure of biochar may facilitate adhesive penetration and mechanical interlocking, potentially contributing to improved stress transfer across the bonded interfaces. Previous reviews have discussed mechanical-property improvements in particle-modified UF-bonded wood composites [1] and the potential of biochar to reinforce polymer composites [46]. These findings provide context for the observed IB response, although differences in resin chemistry, filler characteristics, and processing conditions limit direct comparison with the present UmF-bonded particleboards. In contrast, the relatively larger particle size and highly microporous structure of activated carbon may limit its dispersion within the matrix and reduce the effective contact area between the adhesive and wood particles [4,21,22].

The MOR and MOE results of the particleboard panels are presented in Table 4. All panels exhibited higher MOR and MOE values than those of the control. The most statistically significant improvements in MOR values were observed in the panels produced with 0.5% of AC or BC, with about 36% and 33% higher values than those of the control boards, respectively (*p* ≤ 0.05). Although the increases in MOE values were not statistically significant compared with the control (*p* > 0.05), a general upward trend was observed for the biochar-modified boards. In the panels containing AC, a decreasing trend in MOR and MOE was observed as loading increased, suggesting that higher AC content adversely affects bending performance. In contrast, BC boards showed consistently improved MOR values across all loadings, with relatively stable performance compared to AC. The incorporation of carbon-based fillers can improve stress distribution and enhance the transfer of applied loads between wood particles, thereby enhancing the integrity of the bonded network [1,47]. Similar improvements in the mechanical performance of wood-based composites containing activated carbon and other carbonaceous fillers have been reported in previous studies [4,48]. The loading-dependent MOR response may be associated with the filler–matrix interactions discussed above. At low scavenger loading, the particles may contribute to stress transfer without substantially disrupting the adhesive phase, whereas higher loadings may introduce heterogeneities within the bonded structure. However, particle dispersion and agglomeration were not directly characterized, and these mechanisms should therefore be considered possible explanations rather than experimentally confirmed effects [22,49]. One possible explanation for the observed stiffness response is that a more uniform particle distribution could reduce local stress concentrations and facilitate stress transfer within the composite.

Unlike MOR, which reflects the ultimate load-bearing capacity, MOE is primarily governed by the rigidity of the composite structure under small deformations. The observed improvement in MOE may therefore be attributed to the incorporation of rigid carbon-based particles within the adhesive matrix, which can enhance resistance to elastic deformation and contribute to a stiffer composite structure [1,15]. Similar effects have been reported for biochar and biomass-derived fillers incorporated into polymer and adhesive systems, where improved filler dispersion enhanced mechanical performance [50]. The decline in MOE with increasing AC loading could reflect changes in particle dispersion or adhesive distribution. However, agglomeration and adsorption of adhesive components were not measured, so their contribution to the observed trend remains uncertain.

### 3.4. Thickness Swelling

**Figure 5:**
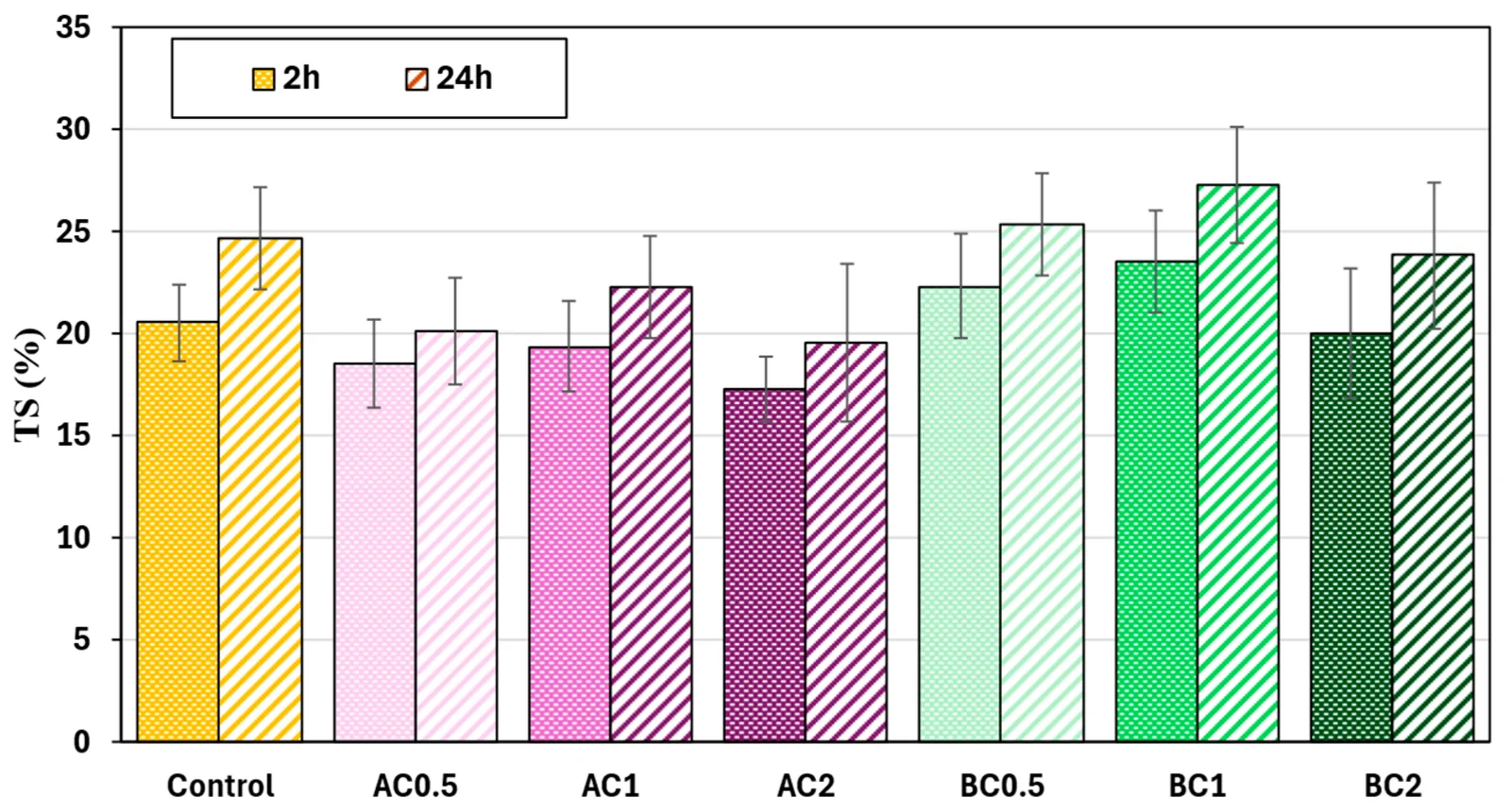
Comparison of TS values of particleboard panels with and without scavengers after 2 h and 24 h immersion in water.

The thickness swelling (TS) results of the modified particleboards after immersion in water are presented in Table 5 and Figure 5. The values after 24 h immersion illustrated that the incorporation of AC significantly reduced TS compared to the control board, with the greatest reduction observed for AC2 (approximately 21%) and AC0.5 (approximately 18%). These reductions were statistically significant (*p* ≤ 0.05), indicating a clear improvement in dimensional stability. In contrast, BC boards generally exhibited higher TS values than the control, particularly at intermediate loadings, where swelling increased significantly (*p* ≤ 0.05). BC2 showed a numerically lower TS than the control; however, the difference was not statistically significant (*p* > 0.05). The improved dimensional stability of AC-modified boards may be attributed to the high surface area and fine porous structure of activated carbon, which can contribute to a denser composite structure, reduce the accessibility of water to internal voids and capillary pathways, and adsorb water. Similar improvements in the physical properties of wood-based composites containing activated carbon have been reported in previous studies [51,52]. The higher mean TS observed for BC0.5 and BC1 may reflect differences in water accessibility, surface chemistry, or adhesive distribution. However, the available BET surface areas alone do not establish the pore-size distributions or water affinity of the additives. The slightly lower mean TS of BC2 could reflect changes in void filling or adhesive distribution, but its difference from the control was not statistically significant in the reported analysis. These explanations therefore remain hypotheses requiring further characterization.

The loading-dependent mechanical response can be compared with that reported by Kawalerczyk et al. [22] for UF-bonded particleboards containing coconut-shell activated carbon. They observed approximately 25% higher internal bond strength at 0.75–1.0% AC, whereas increasing the loading to 1.5% returned the strength to approximately the control level. In the present study, AC did not produce a consistent increase in mean IB, while BC1 showed an approximately 18% increase. Differences in additive characteristics, adhesive composition, and manufacturing conditions limit direct quantitative comparisons.

Across the mechanical properties and thickness swelling measurements, exploratory diagnostic tests detected no departures from residual normality (Shapiro–Wilk, *p* = 0.3996–0.9761) or homogeneity of variance (median-based Levene, *p* = 0.4061–0.7189).

### 3.5. Formaldehyde Emission

The formaldehyde emission results of the particleboards are presented in Table 6. The measured values ranged from 0.47 to 0.54 mg/L. The incorporation of AC generally reduced formaldehyde emissions compared with the control, whereas BC reduced emissions at 0.5% and 1.0% loading but resulted in a slight increase at 2.0% loading. The most pronounced reductions were observed for BC0.5 and BC1, with decreases of approximately 10% and 11%, respectively, compared with the control. In contrast, AC-containing boards showed more moderate reductions, with a maximum decrease of approximately 8% at the lowest level (AC0.5). At higher percentages, the effectiveness of AC decreased, and a negligible reduction was observed for AC2. Although the absolute differences were modest, the mean formaldehyde release was approximately 10–11% lower for BC0.5 and BC1 than for the control. These relative reductions suggest potential for emission mitigation. Kawalerczyk et al. [22] reported an approximately 16% reduction in formaldehyde content at AC loadings of 1.0–1.5%, whereas the largest mean reduction for AC in the present study was approximately 8% at 0.5% loading. BC0.5 and BC1 showed mean reductions of approximately 10–11%. These results indicate different loading responses across the two studies. However, Reference [22] determined formaldehyde content using the EN 120 perforator method, whereas the present study measured formaldehyde release using the procedure described in Section 2.4. The percentage reductions therefore provide contextual comparisons rather than a direct ranking of scavenging effectiveness.

**Figure 6:**
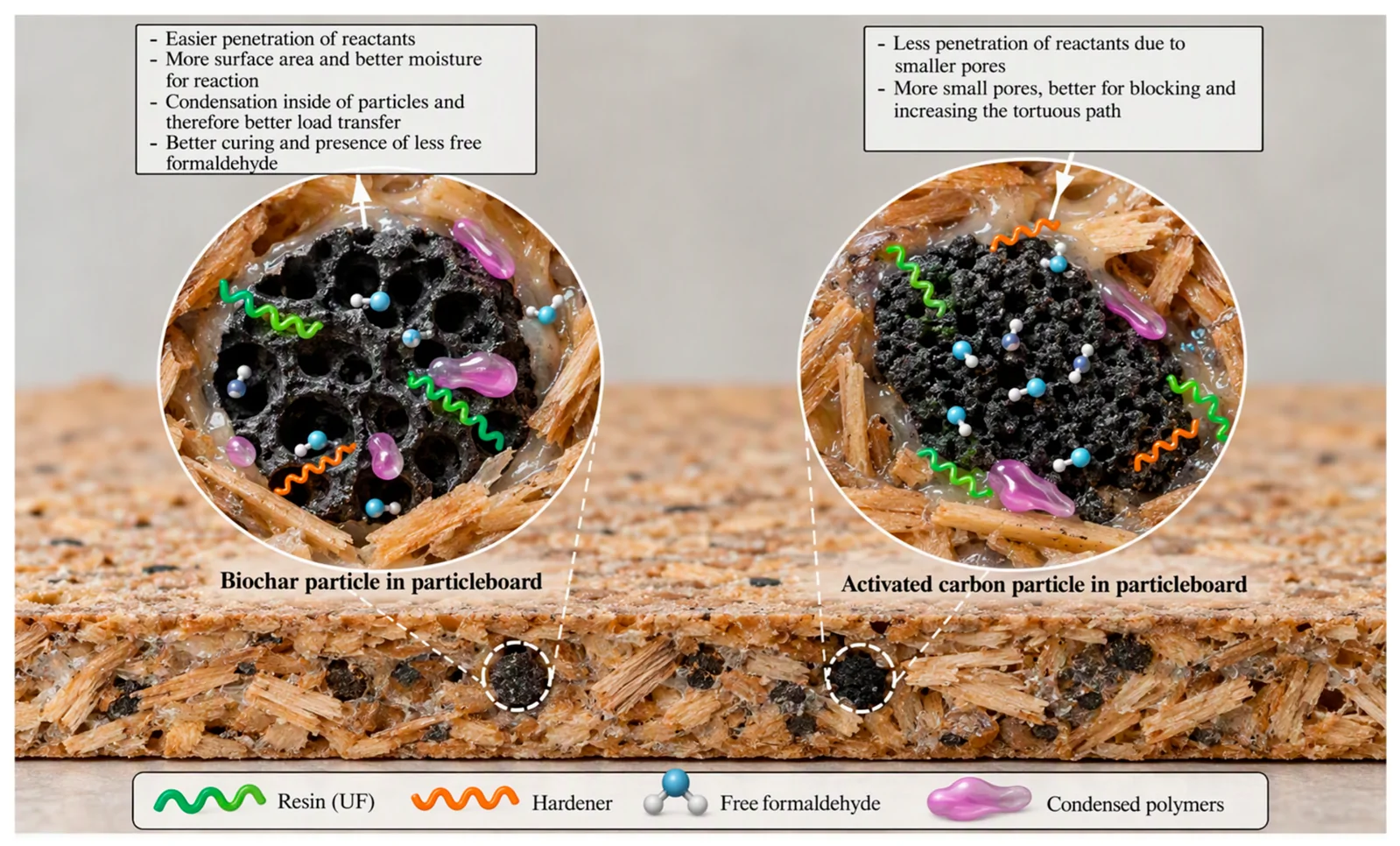
Proposed pathways by which AC and BC may influence formaldehyde release from UmF-bonded particleboards, including physical adsorption and possible effects on adhesive distribution and curing.

Figure 6 illustrates possible pathways through which AC and BC may influence formaldehyde release from UmF-bonded particleboards. Both additives may physically adsorb formaldehyde within accessible pores, reducing the fraction available for release. However, adsorption within the adhesive–wood matrix depends on pore accessibility and surface chemistry, rather than specific surface area alone. Adhesive components may occupy or block pores, limiting the adsorption capacity available after incorporation into the board [3,17,18].

The porous structure of BC may also influence adhesive penetration and the local distribution of adhesive components. These interactions could affect adhesive curing and, consequently, the amount of residual formaldehyde available for release. This provides a possible explanation for the observed emission trends, but curing enhancement cannot be established from the emission measurements alone. The slight increase observed for BC2 may reflect competing effects, such as changes in adhesive distribution or curing at higher loading; however, the present data cannot distinguish these explanations from experimental variability. Figure 6 should therefore be interpreted as a conceptual illustration of possible mechanisms rather than direct evidence of their occurrence.

### 3.6. Environmental Assessment

The environmental performance of the particleboards was initially assessed by evaluating the impacts associated with the production of AC and BC, and then the factors influencing the panels as a whole. As presented in Table 7, the AC exhibits higher environmental burdens across most impact categories compared to BC. In particular, the production of AC shows substantially higher values in global warming potential (GWP), fossil fuel depletion, human toxicity, and aquatic ecotoxicity. The higher modeled impacts of AC are consistent with the findings of Shaheen et al. [53], who reported greater energy demand for activated carbon production than for biochar production. Their activated carbon production route included additional steam activation. However, the contribution of individual activation steps was not separately evaluated in the present study.

However, this trend was not observed in all impact categories. BC showed higher values for terrestrial ecotoxicity and photochemical oxidation than AC (Table 7), indicating that the environmental advantage of BC is impact-category dependent. These differences may be related to the specific feedstock, pyrolysis conditions, energy sources, and emission inventories used in the LCA. Therefore, the results should be interpreted as a comparison of the modeled production systems rather than as an inherent environmental superiority of one material under all conditions.

The baseline environmental impacts of the particleboards were similar across formulations (Table 8). For example, global warming potential ranged from approximately 212.69 to 213.78 kg CO_2_ eq per m^3^, a difference of about 0.5% relative to the control. Although the impacts per unit mass of AC and BC differed considerably in several categories (Table 7), their low inclusion rates limited their contribution to the total board impacts. The common production and transportation contributions, together with the largely similar material inventories, therefore reduced the differences between formulations. These results indicate that the lower modeled production impacts of BC in most categories did not translate into a substantial reduction in the baseline impacts of the finished boards. A supplementary normalization was therefore used to explore how differences in technical performance could affect annualized impacts. The performance index combined IB, MOR, and inverse TS ratios with equal weighting, and the resulting assumed service lives are presented in Table 9. BC2 had the highest index, corresponding to 34.86 years, followed by AC1 at 34.02 years. Table 10 presents the raw and annually normalized impacts of the control board, while Table 11 isolates the effect of service-life normalization under an equal-raw-impact assumption. Under this assumption, BC2 showed the largest reduction in annualized impacts (13.94%), followed by AC1 (11.82%). The remaining formulations showed reductions of 7.86–10.55%. These results indicate that the ranking depends on the individual formulation rather than scavenger type alone. The calculated service lives and environmental reductions are conditional on the assumed model and should not be interpreted as experimentally established durability benefits.

Raw results refer to 1 m^3^ of particleboard. Annually normalized results were obtained by dividing the raw impacts by the assumed control service life of 30 years and are expressed in the corresponding impact unit per year for 1 m^3^ of particleboard.

Table 12 presents the percentage changes in environmental impacts resulting from ±50% variation in the production impacts assigned to BC and AC, relative to the baseline result for each formulation.

The ±50% variation in BC and AC production impacts resulted in relatively small changes in the total environmental impacts of the boards. The largest response occurred for photochemical oxidation in BC2.0 (±2.121%), while changes in all other impact categories remained below ±0.4%. Global warming impacts varied by up to ±0.232% for AC-containing boards and ±0.105% for BC-containing boards. These results indicate limited sensitivity of the total board impacts to the tested variations, although they do not eliminate uncertainty associated with the representativeness of the production datasets.

The weighting sensitivity analysis showed that the ranking of formulations depended on the relative importance assigned to the selected properties (Table 13). BC2 had the highest assumed service life under equal weighting and under the IB- and MOR-emphasis scenarios, whereas AC0.5 ranked highest when TS was emphasized. All modified formulations retained higher performance indices than the control under the tested weighting scenarios. However, these results reflect an exploratory comparison of measured technical properties and do not establish actual service-life extensions.

The findings of this study should be interpreted within the limitations of the experimental design and modeling assumptions. The investigation was conducted using a specific UmF adhesive, wood-particle source, manufacturing conditions, and AC and BC loading range; therefore, the results may not be directly generalizable to other particleboard systems. Furthermore, the proposed mechanisms for particle dispersion, adhesive penetration, adsorption, and curing were inferred from experimental trends and the literature rather than directly characterized. The service-life normalization is also a comparative model based on assumed relationships between IB, MOR, TS, and panel service life, rather than experimentally measured long-term durability. Equal weighting of these properties was adopted as a neutral assumption, although alternative weighting factors could influence the calculated ranking of the panels. Future studies should validate the proposed mechanisms through microstructural and curing characterization and evaluate the service-life model using long-term durability data. Nevertheless, the present case study demonstrates the potential value of jointly considering formaldehyde emission, technical performance, and environmental impacts when evaluating carbon-based scavengers for particleboard production.

## 4. Conclusions

The results indicate that AC and BC should be evaluated according to the intended performance requirements of UmF-bonded particleboards, because neither additive consistently improved all measured properties. The FTIR spectra for AC and BC showed that the stronger O–H and aromatic/conjugated bands in activated carbon relative to biochar suggested that differences in surface polarity and functional-group distribution may contribute to their distinct interactions with UmF, although FTIR alone cannot confirm a specific bonding or curing mechanism. The DSC results indicate that activated carbon and biochar modify the apparent, heating-rate-dependent thermal response of the UmF adhesive, but the effects vary with additive type and loading and do not establish a consistent cure-accelerating or cure-retarding trend. The observed response to BC was not proportional to loading; increasing its content to 2.0% did not improve formaldehyde control. Thus, a higher carbon addition cannot be assumed to provide greater scavenging effectiveness. Within the investigated formulations, BC1 combined higher mean internal bond strength with lower mean formaldehyde release, whereas AC2 provided the greatest reduction in thickness swelling. These trends support selecting formulations through a balance of emission control, bonding performance, and dimensional stability rather than a single property.

The environmental assessment likewise requires a distinction between baseline impacts and performance-based projections. The small differences in baseline impacts per cubic metre indicate that incorporating these low additive contents did not substantially alter the modeled production impacts of the boards. The approximately 14% lower annualized impacts calculated for BC2 arose under the assumed service-life model, equal property weighting, and equal-raw-impact assumption; they do not demonstrate measured durability gains. Moreover, BC2’s favorable model ranking coincided with slightly higher mean formaldehyde release, illustrating why this ranking alone cannot identify the preferred formulation.

These findings support further investigation of low-dose carbon additives while highlighting the limitations of selecting formulations solely through emission measurements or modeled environmental performance. Given the limited independent board replication, the experimental trends require confirmation across independently manufactured boards. Long-term durability measurements are also needed before the projected service-life benefits can be treated as demonstrated environmental advantages.

## Figures and Tables

**Table 1:** Details of the panel compositions using activated carbon and biochar scavengers *.

Panel Code	Adhesive	Scavenger Type	
		Activated Carbon (%)	Biochar (%)
Control	UmF	-	-
AC0.5	UmF	0.5	-
AC1	UmF	1	-
AC2	UmF	2	-
BC0.5	UmF	-	0.5
BC1	UmF	-	1
BC2	UmF	-	2

* Two panels were prepared per composition type.

**Table 2:** Ecoinvent datasets used in the life cycle inventory.

Material/Process	Ecoinvent Dataset	Geography	Unit
Activated carbon	Market for activated carbon, granular	GLO	kg
UmF adhesive	Melamine–urea–formaldehyde adhesive production	GLO	kg
Wood chips	Wood chips production, softwood, at sawmill	Europe without Switzerland	kg
Tap water	Market for tap water	Europe without Switzerland	kg
Biochar	Charcoal production	GLO	kg
Electricity	Market for electricity, high voltage	SE	kWh
Transportation	Market for transport, freight, light commercial vehicle	Europe without Switzerland	tkm

GLO: global; SE: Sweden; tkm: tonne-kilometre, representing the transport of one metric tonne of goods over one kilometre.

**Table 3:** Average IB values of particleboards containing AC and BC scavengers, and their relative change to the control.

	Control	AC0.5	AC1	AC2	BC0.5	BC1	BC2
IB (MPa)	0.60 ±0.08	0.46 ±0.11	0.66 ±0.13	0.54 ±0.10	0.59 ±0.06	0.71 ±0.11	0.66 ±0.07
Change relative to control (%)	-	−22.92	9.26	−10.00	−2.08	18.33	10.42
*p*-value	-	0.01	0.28	0.15	0.33	0.05	0.11

**Table 4:** MOR and MOE of particleboards containing AC and BC scavengers, together with percentage change relative to the control.

	Control	AC0.5	AC1	AC2	BC0.5	BC1	BC2
MOR (MPa)	5.6 ±1.43	7.6 ±0.64	6.7 ±0.59	6.1 ±1.18	7.5 ±0.69	7.0 ±0.82	7.6 ±0.45
Change relative to control (%)	-	35.8	20.0	9.2	33.0	24.7	34.5
*p*-value	-	0.05	0.45	0.91	0.03	0.25	0.65
MOE (MPa)	1306.7	1625.4	1526.8	1376.6	1415.4	1491.2	1518.6
Change relative to control (%)	-	24.4	16.8	5.3	8.3	14.1	16.2
*p*-value	-	0.13	0.36	0.46	0.27	0.72	0.13

**Table 5:** TS of particleboards containing AC and BC scavengers after 24 h immersion in water, and their respective changes compared to the control panels.

	Control	AC0.5	AC1	AC2	BC0.5	BC1	BC2
Swelling (%)	24.68 ±1.92	20.15 ±2.17	22.25 ±2.22	19.53 ±1.55	25.35 ±2.54	27.30 ±2.51	23.81 ±3.22
Change relative to control (%)	-	−18.35	−9.85	−20.88	2.70	10.60	−3.51
*p*-value	-	0.001	0.065	0.002	0.993	0.040	0.536

**Table 6:** Formaldehyde emissions of particleboards containing AC and BC scavengers and their relative changes compared with the control.

	Control	AC0.5	AC1	AC2	BC0.5	BC1	BC2
Formaldehyde Emission (mg/L)	0.52	0.48	0.50	0.51	0.47	0.47	0.54
Average Change %	-	−8.2	−5.2	−1.8	−10.0	−11.0	2.7

**Table 7:** LCA comparison of 1 ton of AC and BC production.

Impact Category	Unit	AC	BC
Abiotic depletion	kg Sb eq	1.59 × 10^−3^	4.87 × 10^−4^
Abiotic depletion (fossil fuels)	MJ	3.70 × 10^4^	2.70 × 10^3^
Global warming (GWP100a)	kg CO_2_ eq	3.30 × 10^3^	1.50 × 10^3^
Ozone layer depletion (ODP)	kg CFC-11 eq	1.63 × 10^−5^	1.89 × 10^−6^
Human toxicity	kg 1,4-DB eq	1.37 × 10^3^	1.10 × 10^2^
Freshwater aquatic ecotox.	kg 1,4-DB eq	1.00 × 10^3^	6.80 × 10^1^
Marine aquatic ecotoxicity	kg 1,4-DB eq	3.65 × 10^6^	2.02 × 10^5^
Terrestrial ecotoxicity	kg 1,4-DB eq	9	23
Photochemical oxidation	kg C_2_H_4_ eq	0.9	8.85
Acidification	kg SO_2_ eq	19.65	1.28
Eutrophication	kg PO_4_ eq	5.33	0.62

Sb: antimony; CFC-11: trichlorofluoromethane; 1,4-DB: 1,4-dichlorobenzene; eq: equivalents.

**Table 8:** Baseline environmental impacts of the control and AC- and BC-containing particleboards per 1 m^3^ of board, before service-life normalization.

Impact Category	Control	AC0.5	AC1.0	AC2.0	BC0.5	BC1.0	BC2.0
Abiotic depletion	1.59 × 10^−3^	1.59 × 10^−3^	1.59 × 10^−3^	1.59 × 10^−3^	1.59 × 10^−3^	1.59 × 10^−3^	1.59 × 10^−3^
Abiotic depletion (fossil fuels)	2398.51	2402.56	2405.22	2410.85	2399.81	2400.11	2400.11
Global warming (GWP100a)	212.69	213.04	213.28	213.78	212.89	213.01	213.20
Ozone layer depletion (ODP)	4.33 × 10^−6^	4.33 × 10^−6^	4.33 × 10^−6^	4.33 × 10^−6^	4.33 × 10^−6^	4.33 × 10^−6^	4.33 × 10^−6^
Human toxicity	288.90	289.06	289.16	289.37	288.96	288.97	288.97
Freshwater aquatic ecotox.	141.52	141.64	141.71	141.86	141.57	141.58	141.57
Marine aquatic ecotoxicity	235,188.42	235,561.27	235,826.89	236,381.74	235,288.12	235,310.58	235,309.98
Terrestrial ecotoxicity	2.84	2.85	2.85	2.85	2.85	2.85	2.85
Photochemical oxidation	0.06	0.06	0.06	0.06	0.06	0.06	0.06
Acidification	0.81	0.81	0.81	0.82	0.81	0.81	0.81
Eutrophication	0.34	0.34	0.34	0.34	0.34	0.34	0.34

**Table 9:** Performance indices and estimated relative service-life values of the particleboards based on their technical properties.

	IB (MPa)	MOR (MPa)	TS 24 h (%)	S_IB_	S_MOR_	S_TS_	W_IB_	W_MOR_	W_TS_	PI	SL (Years) *
Control	0.60	5.6	24.7	1.0	1.0	1.0	1/3	1/3	1/3	1.00	30.00
AC0.5	0.46	7.6	20.2	0.8	1.4	1.2				1.12	33.54
AC1	0.66	6.7	22.3	1.1	1.2	1.1				1.13	34.02
AC2	0.54	6.1	19.5	0.9	1.1	1.3				1.08	32.56
BC0.5	0.59	7.5	25.4	1.0	1.3	1.0				1.09	32.83
BC1	0.71	7.0	27.3	1.2	1.2	0.9				1.11	33.34
BC2	0.66	7.6	23.8	1.1	1.3	1.0				1.16	34.86

* SL represents an assumed service life used for comparative normalization, not a measured lifespan.

**Table 10:** Raw and annually normalized LCA results for the control.

Impact Category	Unit	Control Board (Raw Result)	Control Board (Normalized)
Abiotic depletion	kg Sb eq	1.59 × 10^−3^	5.31 × 10^−5^
Abiotic depletion (fossil fuels)	MJ	2398.51	79.95
Global warming (GWP100a)	kg CO_2_ eq	212.69	7.09
Ozone layer depletion (ODP)	kg CFC-11 eq	4.33 × 10^−6^	1.44 × 10^−7^
Human toxicity	kg 1,4-DB eq	288.90	9.63
Freshwater aquatic ecotox.	kg 1,4-DB eq	141.52	4.72
Marine aquatic ecotoxicity	kg 1,4-DB eq	235,188.42	7839.61
Terrestrial ecotoxicity	kg 1,4-DB eq	2.84	0.09
Photochemical oxidation	kg C_2_H_4_ eq	0.06	0.002
Acidification	kg SO_2_ eq	0.81	0.03
Eutrophication	kg PO_4_eq	0.34	0.01

**Table 11:** Relative changes in annually normalized environmental impacts of scavenger-containing particleboards compared with the control board.

Scavenger-Containing Board	Relative Change Due to Service-Life Normalization (%)
AC0.5	−10.55
AC1	−11.82
AC2	−7.86
BC0.5	−8.62
BC1	−10.02
BC2	−13.94

**Table 12:** Percentage changes in particleboard environmental impacts resulting from ±50% variation in BC or AC production impacts, with all other inputs held constant. Values are relative to each formulation’s baseline; ± denotes equal-magnitude increases and decreases.

Impact Category	AC0.5	AC1.0	AC2.0	BC0.5	BC1.0	BC2.0
Abiotic depletion	±0.004	±0.007	±0.015	±0.001	±0.002	±0.005
Abiotic depletion (fossil fuels)	±0.058	±0.115	±0.229	±0.004	±0.008	±0.017
Global warming (GWP100a)	±0.058	±0.116	±0.232	±0.026	±0.053	±0.105
Ozone layer depletion (ODP)	±0.014	±0.028	±0.056	±0.002	±0.003	±0.007
Human toxicity	±0.018	±0.035	±0.071	±0.001	±0.003	±0.006
Freshwater aquatic ecotox.	±0.026	±0.052	±0.104	±0.002	±0.004	±0.007
Marine aquatic ecotoxicity	±0.058	±0.116	±0.232	±0.003	±0.006	±0.013
Terrestrial ecotoxicity	±0.011	±0.022	±0.045	±0.030	±0.060	±0.119
Photochemical oxidation	±0.056	±0.112	±0.224	±0.548	±1.081	±2.121
Acidification	±0.091	±0.180	±0.360	±0.006	±0.012	±0.024
Eutrophication	±0.059	±0.117	±0.233	±0.007	±0.014	±0.027

**Table 13:** Sensitivity of model-derived assumed service life (years) to the weighting of internal bond strength (IB), modulus of rupture (MOR), and thickness swelling (TS). Equal weighting assigns 1/3 to each property. Each emphasis scenario assigns a weight of 0.50 to the selected property and 0.25 to each of the other two properties.

Formulation	Equal Weights	IB Emphasis	MOR Emphasis	TS Emphasis
Control	30	30	30	30
AC0.5	33.5	30.9	35.3	34.3
AC1	34	33.7	34.5	33.8
AC2	32.6	31.2	32.6	33.9
BC0.5	32.8	32	34.6	31.9
BC1	33.3	33.9	34.4	31.8
BC2	34.9	34.4	36.2	33.9

## Data Availability

The original contributions presented in this study are included in the article. Further inquiries can be directed to the corresponding author.

## Abbreviations

The following abbreviations are used in this manuscript:

AC	Activated carbon
BC	Biochar
UmF	Urea–melamine–formaldehyde
UF	Urea–formaldehyde
IB	Internal bond strength
MOR	Modulus of rupture
MOE	Modulus of elasticity
TS	Thickness swelling
LCA	Life cycle assessment
LCI	Life cycle inventory
LCIA	Life cycle impact assessment
FU	Functional unit
PI	Performance index
RSL	Reference service life
SL	Assumed service life
GWP	Global warming potential
ODP	Ozone depletion potential
